# The promise and perils of humanized mice: workshop report from the 29th scientific conference of the society on NeuroImmune pharmacology (SNIP)

**DOI:** 10.1515/nipt-2025-0014

**Published:** 2025-10-24

**Authors:** Paul W. Denton, Ramesh Akkina, Howard E. Gendelman, Jennifer E. Koblinski, Angela Wahl, Santhi Gorantla

**Affiliations:** Department of Biology, University of Nebraska at Omaha, Omaha, NE, USA; Department of Microbiology, Immunology, and Pathology, Colorado State University, Fort Collins, CO, USA; Department of Pharmacology and Experimental Neuroscience, University of Nebraska Medical Center, Omaha, NE, USA; Department of Pathology, Massey Comprehensive Cancer Center, Virginia Commonwealth University, Richmond, VA, USA; Department of Microbiology, The University of Alabama at Birmingham, Birmingham, AL, USA

**Keywords:** humanized mice, neurology, immunology, pharmacology, infectious disease, cancer

## Abstract

On June 8, 2025, the 29th Scientific Conference of the Society on NeuroImmune Pharmacology (SNIP) hosted a workshop on the *Creation, Care, and Translation of Humanized Mouse for HIV/AIDS Research*. The workshop was convened by the society officers Drs. Howard E. Gendelman and Santhi Gorantla. A series of four presentations provided details about the generation, care and use of humanized mouse models. The presentation titles and presenters were: (i) “Next-Generation Humanized Mouse Models of HIV/AIDS Research” by Dr. Angela Wahl; (ii) “Advancing Humanized Mice Research Through Shared Resources” by Dr. Jennifer Koblinski; (iii) “NeuroHIV Humanized Mouse Models” by Dr. Santhi Gorantla; and (iv) “Studies on HIV Evolution, Latency, and Elite Control in Humanized Mice” by Dr. Ramesh Akkina. The presentations were followed by a discussion with workshop participants led by Dr. Paul W. Denton. Presentation summaries are provided in this report and are followed by questions offered by workshop participants alongside panel responses.

## Summary of Presentations

### “Next-Generation Humanized Mouse Models of HIV/AIDS Research”

#### Dr. Angela Wahl

This presentation covered three main themes. (i) The first theme was to define humanized mice to the audience as immunodeficient mice transplanted with human cells and/or tissues [1] and to briefly overview how to generate two common humanized mouse models for HIV/AIDS research – human CD34 transplant mice and bone marrow/liver thymus (BLT) mice. This was followed by a review of key papers demonstrating the effective recapitulation of HIV transmission and pathogenesis in humanized mice [2], [3], [4], [5], [6], [7], [8], [9] as well as their use in preclinical testing of HIV prevention, treatment, and cure strategies [4], 8], [10], [11], [12], [13], [14], [15]. (ii) The second theme focused on developing germ-free humanized mice to study how resident microbiota influence health and disease [16]. The presence of human immune cells in the peripheral blood and tissues (e.g., spleen, lymph nodes, bone marrow, thymus, liver, lung, and gastrointestinal tract) was confirmed in germ-free humanized mice models. To evaluate the role of resident microbiota in HIV transmission, germ-free humanized mice and humanized mice colonized with resident mouse microbiota and housed in specific pathogen free (SPF) conditions (conventional humanized mice) were exposed rectally to HIV. Data presented during the workshop showed that HIV acquisition was significantly higher in conventional humanized mice. In addition, the levels of HIV-RNA were higher in the peripheral blood and tissues of HIV-infected conventional humanized mice. These data showed that resident microbiota substantially impacts both HIV acquisition as well as HIV replication [16]. There are many potential lines of future investigation that germ-free humanized mice models permit. These include evaluating the role of resident microbiota in HIV persistence, latency reversal, and the efficacy of cure approaches as well as in HIV infection in the CNS. In addition, germ-free mice can be colonized with human microbiota to assess how human resident microbiota and the diversity in microbial composition between people affects HIV infection. (iii) The third theme was to emphasize the utility of leveraging novel humanization methods in the fight against emerging pathogens. In this section, the specific focus was on how immunodeficient mice can be implanted with human lung tissue (humanized lung-only mice or LoM) to facilitate the study of human pathogens that replicate in the respiratory tract (e.g., Middle East respiratory syndrome coronavirus [MERS-CoV], severe acute respiratory syndrome [SARS]-CoV, SARS-CoV-2, Zika virus, human respiratory syncytial virus, human cytomegalovirus, and mycobacteria) [17], 18]. LoM were successfully used to perform preclinical evaluations of an intervention targeting SARS-CoV-2 [18]. The overall conclusion focused on the many humanized mouse models available to researchers and the broad utility of these models to help fight many human diseases.

### “Advancing Humanized Mice Research Through Shared Resources”

#### Dr. Jennifer Koblinski

This presentation explored the pivotal role of core facilities in advancing humanized mouse research, emphasizing collaborative strategies with investigators [19], 20] and technical innovations. It began by outlining various approaches for generating humanized mouse models, highlighting how core facilities can streamline this process by handling model development and validation before the mice are distributed to investigators. This centralized workflow ensures consistency, quality control, and efficiency in preclinical research. A significant portion of the presentation focused on the challenges posed by *Corynebacterium bovis* contamination within mouse facilities. The pathogen’s impact on immunocompromised mice, particularly those used in humanized and patient-derived xenograft (PDX) models, was discussed in depth [21], 22]. The presentation detailed the implementation of a robust surveillance and containment program [23], showcasing how the core facility successfully mitigated the risk of infection and maintained the integrity of its specialized mouse colonies. The discussion then transitioned to the generation of PDX mice using harvested cancerous tissue. While this technique offers powerful insights into personalized cancer therapy, it also presents biosafety risks, including the potential reintroduction of *C. bovis* into the facility. Strategies for minimizing this risk were shared, including tissue handling protocols and facility design considerations. The utility of PDX models was illustrated through clinically relevant examples, demonstrating how tumors derived from individual patients can be engrafted into multiple mice to simulate treatment responses. This approach enables researchers to test various therapeutic combinations and refine clinical decision-making. The presentation referenced key publications [24], [25], [26] that validate the predictive power of PDX models in translational cancer research. Overall, the presentation highlighted the vital role of core facilities not only in technical execution but also in maintaining pathogen-free environments and supporting precision medicine. By incorporating strict quality control, innovative modeling methods, and collaborative frameworks, core facilities play a crucial role in accelerating research and therapeutic development.

### “NeuroHIV Humanized Mouse Models”

#### Dr. Santhi Gorantla

This presentation focused on using humanized mice to replicate human brain diseases in the context of neuroHIV. The presentation discussed modeling HIV-associated neurocognitive disorders (HAND). The presented research highlights serious neurological problems caused by HIV that continue despite effective antiretroviral therapy (ART) [27]. The presentation started with key questions needing further study. These questions included how ongoing low-level HIV replication in the brain leads to clinical signs and symptoms despite ART; whether the central nervous system (CNS) remains as a major viral reservoir contributing to viral rebound after stopping ART; whether neuroinflammation continues in patients on ART; what are the underlying mechanisms of neurocognitive dysfunction during persistent viral infection; and whether ART toxicity plays a role in neurological damage. Another question asked whether HIV infection increases the risk of developing Alzheimer’s disease. Although not all questions were answered, pathways were outlined for how humanized mouse models could help investigate each issue. A general overview of the use of humanized mice in the context of neuroHIV was presented. Most early humanized mouse models face challenges due to the limited presence of human immune cells in the brains of the transplanted mice. To address this, a new model was generated that led to the transgenic expression of human IL-34 [28]. The presence of IL-34 helps differentiate human microglia-like cells in these animals [28]. The presentation concluded with new data showing that HIV-1 neuropathogenesis can be studied alongside Alzheimer’s disease in immunodeficient mice that combine transgenic expression of human IL-34 with modifications to the mouse amyloid precursor protein gene [29].

### “Studies on HIV Evolution, Latency, and Elite Control in Humanized Mice”

#### Dr. Ramesh Akkina

This presentation began with a general overview of advances in humanized mouse research in the context of HIV pathogenesis, treatment and prevention [30], [31], [32], [33], [34]. Expanding the utility of humanized mouse systems to a pair of novel topics was discussed. (i) How HIV-1 and HIV-2 originated/evolved from their non-human primates-derived simian immunodeficiency virus (SIV) ancestor viruses remains a mystery. Humanized mice were initially infected with SIVcpz native to chimpanzees and SIVsm native to sooty mangabeys to evaluate their evolution into HIV-1 and HIV-2 respectively. Subsequently, replicating viruses were serially passaged in humanized mice for several generations to evaluate human adaptive changes. Viral loads were found to be higher with each subsequent transfer. During later passages, helper CD4 T cell decline was also noted. Genetic analysis of the humanized mouse adapted viruses showed many mutations, distributed throughout the genome including gag, env, nef, vpu, vpr, vif and rev genes [35], [36], [37]. Many of these mutations are involved in overcoming human restriction factors and further analysis will likely shed new light on viral adaptation and evolution. Since humanized mice are permissive to SIV infection, they can serve as dual purpose models to study comparative aspects both HIV and SIV using the same system [38]. (ii) While ART is effective in suppressing viral loads to undetectable levels, the latent viral reservoir remains a critical barrier to achieving a complete HIV cure. The rare cells that comprise the HIV reservoir are difficult to study and quantify. However, detection of any remnant ultra-low levels of latently infected cells is critical to confirm a potential cure. The gold standard *in vitro* quantitative viral outgrowth assay (qVOA) has not been fully effective due to the assay’s limited time frame wherein not all dormant viruses are induced [39], [40], [41], [42], [43]. In this regard, the *in vivo* viral outgrowth assay using humanized mice (hmVOA) has proved to be more sensitive as it is able to detect latent virus in many samples previously thought to be negative based on outcomes from the standard qVOA [44], 45]. Among the advantages with the hmVOA are the longer assay time frame as well as the physiological conditions afforded in contrast to the *in vitro* assays. The hmVOA was successfully used to evaluate the cells from the fully cured Berlin and London patients wherein no replication competent virus could be found. Exceptional elite controllers (EEC) of HIV are an extremely rare population of individuals with undetectable viral loads, and who exhibit no latent virus by the standard qVOA [46]. Due to their prolonged virus negative status in the absence of antiretroviral therapy, it is suggested that some of these individuals might be fully cured. Thus, the use of a highly sensitive viral outgrowth assay is necessary to determine the status of their latent viral reservoir in these individuals – if the reservoir still exists. Vast numbers of resting memory CD4+ T cells from multiple EEC subjects were evaluated by the hmVOA to assay for the latent viral reservoir. Replication competent virus was detected from one of the five samples tested whereas infrequent viral blips were detected by qRT-PCR in others. Overall, the humanized mouse *in vivo* assays are likely to assist in further characterization of latent viral reservoirs.

## Discussion

Topics raised during the discussion were broad ranging. They covered both the promise and the pitfalls of utilizing humanized mouse models.

### Promise

The promise of humanized mice models was emphasized. Questions were raised about what can be done with some models.

Q1: How can I know if using a humanized mouse model is appropriate for my research question?

Panel response: Many factors should be considered when using humanized mice. For example: If you are working with a human-specific pathogen, then you should consider using a humanized mouse model for *in vivo* testing of transmission, prevention of transmission, pathogenesis, treatment, or cure. Similarly, if your research involves testing therapeutics against human cancer cells, especially in a personalized or precision medicine context, humanized mouse models can provide valuable insights. Humanized mice are particularly useful when standard mouse models cannot replicate human-specific biological responses. However, not all research questions require them, and selecting the appropriate model depends on whether a given model aligns with your experimental goals. The best approach is to consult with an experienced humanized mouse researcher who can help determine whether an existing model meets your needs or if a custom model is needed. Panel members expressed their willingness to discuss this further and encouraged researchers to reach out early in the planning process.

Q2: How are germ-free humanized mice generated, and how can they be used to specifically study the impacts of the microbiome (particularly, the gut microbiome) on HIV disease acquisition and pathogenesis?

Panel response: Generating and using germ-free humanized mice requires specialized facilities and equipment. These are necessary to keep mice germ-free as well as to facilitate the required constant surveillance of the animals’ germ-free status. Not all institutions have access to gnotobiotic facility or the necessary equipment, so it may be best to collaborate with investigators experienced in working with such systems and to discuss plans early with your veterinary staff. To our knowledge, there are no commercially available germ-free strains of severely immunodeficient mice. Therefore, the first step in generating germ-free humanized mice was to rederive immunodeficient mouse strains as germ-free. This can be achieved through sterile-embryo transfer or caesarean (C)-section in partnership with a gnotobiotic facility. Colonies of germ-free immunodeficient mice are then maintained in germ-free gnotobiotic isolators. The humanization process must be performed under germ-free conditions. For generating humanized mouse models involving the transplantation of human CD34+ stem cells, mice need to be preconditioned using either irradiation or chemical myeloablation prior to cell transplant. The irradiation of germ-free mice requires placing them in a sterile irradiation chamber before removing them from the isolator. Mice must be kept in the irradiation chamber throughout the procedure. For models involving surgical tissue implantation, a germ-free surgical isolator is essential. Germ-free humanized mice can be used to study the impact of HIV infection in the absence of microbiota. In addition, germ-free humanized mice can be selectively colonized with mouse or human microbiota to evaluate their impact on HIV infection.

Q3: The PDX models are a valuable resource for identifying effective drugs for cancer patients. How do you choose an immunodeficient mouse strain and where do you implant the patient tumor tissue?

Panel response: Selecting the right immunodeficient mouse strain for PDX modeling depends on the tumor type and research goals. Commonly used strains include NOD scid mice that are also deficient in common gamma chain signaling (e.g., NSG and NOG mice) as well as NOD Rag mice that are deficient in common gamma chain signaling (e.g., NRG mice). These and similar strains of mice lack key mouse-derived immune components such that they can support robust engraftment of human tumors. Tumor implantation sites also vary based on study design. Subcutaneous implantation is widely used for ease of monitoring tumor growth and accessibility. However, orthotopic implantation (i.e., the placing the tumor in the mouse organ that is the same as the human organ of origin; e.g., human liver tumor implanted into the mouse liver) can better mimic the tumor’s native microenvironment and metastatic behavior – providing more clinically relevant data. Panelists emphasized that strain selection and implantation method should align with the research question, whether it involves drug efficacy, tumor progression, or metastasis. Working with a core facility or experienced PDX researcher can help refine these choices and ensure model fidelity.

Q4: How is it that you can use humanized mice to develop a more sensitive viral outgrowth assay (VOA) to better understand the impacts of HIV cure-related interventions?

Panel response: While the *in vitro* qVOA has been useful for measuring the latent viral reservoir in general, this technique often fails to detect the ultra-low levels – as seen in cases like the Boston patients wherein viral rebound was eventually observed [47]. The hmVOA proved to be more sensitive due to a physiological *in vivo* setting and a longer monitoring period of up to 10 weeks which allows more time for viral reactivation than in the conventional qVOA. Since it is costly, hmVOA is recommended when a sterilizing HIV cure is suspected to have been achieved. This method could serve as an alternative to analytical treatment interruption.

Q5: What are some of the newest and most effective humanized mouse models? What are some research applications for these systems?

Panel response: The development of humanized mouse models for studying infectious, degenerative, inflammatory, and cancerous diseases has progressed rapidly. A key focus is on realizing that true humanization can be developed in a mouse. In this context, a truly human immune system means bioengineering a comprehensive (including mature T and B lymphocytes) and systemically functional human immune system (including in lymph nodes and the thymus). Achieving full immune function within lymph nodes of humanized mice (e.g., somatic hypermutation and affinity maturation) has been challenging because follicular dendritic cells are not hematopoietic in origin. However, there have been major advances in this field [48], [49], [50]. Mice with such a complete human immune system have been referred to as THX mice in the literature, and they have been reported to produce neutralizing antibody responses and recapitulate human autoimmune conditions [50]. With these mice available, new possibilities open for even more robust *in vivo* studies on vaccines, cancer, and autoimmunity that closely reflect human conditions. To date, some insightful discoveries related to systemic lupus and COVID-19 have been reported using these mice [50]. Looking ahead, it may be possible to engineer humanized mice with full immune function in their lymph nodes combined with the engagement of specific human cells with *bona fide* human thymic epithelia (as in BLT mice or other humanized mouse models where human thymus is implanted under the kidney capsule of immunodeficient mice). Further engineering such mice to express HLA or other transgenes on mouse cells opens a panorama of opportunities to recapitulate human disease conditions *in vivo* while simultaneously unraveling complex disease mechanisms.

Q6: What are the benefits of working with a core facility or purchasing validated humanized mice from a vendor versus generating humanized mice in your own lab?

Panel response: Working with a core facility or purchasing validated humanized mice from a vendor offers several advantages over generating them in-house. These models require specialized expertise, infrastructure, and significant time to develop and validate. By obtaining mice from a core or vendor, researchers can bypass the complex and costly steps of bioengineering, engraftment, and quality control, allowing them to focus directly on experimental design and data collection. This approach is especially beneficial for labs using a limited number of humanized mice or conducting short-term studies. It also eliminates the need to establish and maintain a dedicated facility, which may not be cost-effective for occasional use. Additionally, core facilities often provide technical support, model validation data, and opportunities for collaboration. Panelists encouraged researchers to reach out early to experienced humanized mouse teams to discuss feasibility, model selection, and potential partnerships. This can help ensure the chosen model aligns with the research question and experimental goals.

### Pitfalls

Some challenges associated with using humanized mice models were highlighted during the presentations. These challenges, along with others were discussed in response to questions raised by workshop participants.

Qa: Why do people refer to humanized mouse models in the plural? Are most humanized mouse models essentially the same?

Panel response: The term “humanized mouse models” is used in the plural because there is no single, standardized model. The phrase has become broad and often ambiguous. It is now applied to a wide range of mice with varying levels of human biological components. For example, mice expressing a single human transgene or colonized with a human-derived microbiome are sometimes called “humanized”, even though they do not harbor human cells. Humanized models, especially as defined for this workshop, involve immunodeficient mice engrafted with human cells or tissues, such as immune cells or tumors. These models differ in how they are engineered, the human elements they contain, and the mouse strains used. Importantly, the scientific community has not yet established a consistent framework or terminology to define and distinguish these models. Panelists emphasized the importance of carefully reviewing the methods section of any study to understand what type of model was used. Recognizing key distinctions is crucial for accurate data interpretation and proper experimental planning. In short, not all “humanized” mice are the same and selecting the right model depends on the specific research question.

Qb: What special equipment is needed to generate humanized mice? How does this change when generating germ-free humanized mice?

Panel response: Humanized mice are generated using highly immunodeficient strains of mice. These mice are extremely vulnerable to infection and must be housed under sterile conditions. All caging, bedding, chow, and water (often acidified) should be sterilized. All handling of mice occurs in a biosafety cabinet with personnel wearing proper personal protective equipment (PPE) (e.g. Tyvek gowns/sleeves, gloves, face mask, hair bonnet, shoe covers). Supplies must also be disinfected prior to entry into the biosafety cabinet. Facility workflows must prevent cross-contamination from rooms housing immunocompetent mice. When generating germ-free humanized mice, the requirements are even more stringent. These mice must be housed in germ-free isolators, and all materials entering the isolator must be sterilized and validated as germ-free. Specialized equipment, such as autoclaves, sterile transfer ports, and isolator-compatible tools, is essential. Maintaining germ-free status requires rigorous monitoring and facility design tailored to prevent microbial exposure. Panelists emphasized that both models need significant infrastructure and expertise, and researchers should consider collaborating with core facilities to ensure proper handling and reduce risks.

Qc: How do you prevent the introduction of non-cancer-related pathogens when implanting human tumor tissue into immunodeficient mice for PDX studies?

Panel response: Preventing pathogen transmission during PDX model generation is essential due to the extreme vulnerability of immunodeficient mice. Panelists emphasized the importance of rigorous tissue screening and aseptic technique. While tumor tissue obtained directly from the operating room under sterile conditions is generally pathogen-free, samples that have been passaged through mice must be re-tested for both human and murine pathogens prior to re-implantation into immunodeficient mice. All procedures should be conducted in biosafety cabinets using sterile instruments and full PPE, including Tyvek gowns/sleeves, gloves, face masks, hair bonnets, and shoe covers. Supplies and surfaces must be thoroughly disinfected, and workflows should be designed to minimize exposure time and prevent cross-contamination. Panelists also mentioned that partnering with a core facility experienced in PDX generation can help ensure proper biosafety practices, validated protocols, and reliable pathogen screening. This approach reduces risks and maintains the integrity of both the model and the data generated.

Qd: Some immunodeficient mice are transplanted with mature human peripheral blood cells. These models are sometimes referred to as hu-PBL or hu-PBMC mice. Why can these models be particularly challenging to work with, especially in the context of neuroimmunology?

Panel response: The use of hu-PBL or hu-PBMC mice in translational research more quickly bridges gaps between preclinical studies and clinical applications. These are “quick” platforms used to help develop new therapies, screen interventions, and possibly elucidate the actions of human immune cells in disease processes. The key limitation in these models is that they very rapidly develop of graft-versus-host disease (GVHD) which shortens experimental windows and confounds data interpretations [51].

Qe: Were there any specific challenges that had to be overcome to allow the simian immunodeficiency virus (SIV) to grow in human cells in humanized mice?

Panel response: To our surprise, both the chimpanzee SIVcpz (the HIV-1 progenitor) and sooty mangabey derived SIVsmm (the HIV-2 progenitor) viruses readily infected humanized mice and caused persistent viremia within a week after initial exposure by the intraperitoneal route of infection. Thus, it became clear that these viruses inherently have the capacity to infect humans during accidental exposures.

Qf: Do humanized mouse models developed through transplantation of human cells or tissues show signs of GVHD, and are there strategies to reduce this risk?

Panel response: GVHD-like sequelae are certainly possible when conducting research using mice that are human-mouse chimeras, particularly those involving human immune cell engraftment. Although such outcomes are sometimes viewed positively [52], researchers typically prefer to avoid GVHD-like outcomes whenever possible. This is particularly true when long-term experimental observations are needed. Several strategies can help reduce the risk of GVHD developing. One is selecting an appropriate immunodeficient mouse strain, as some strains are more permissive for human cell or tissue engraftment and have lower incidences of GVHD [53]. Matching of the sex of the human donor and the recipient mouse may also influence GVHD development [54], 55]. Importantly, GVHD-like symptoms may be confused with infections such as *Corynebacterium bovis*, which causes skin irritation and scaling in immunodeficient mice. Recent studies suggest *C. bovis* may worsen GVHD-like symptoms [22]. Therefore, it is essential to ensure that the facility is free of *C. bovis* if such symptoms occur. Panelists emphasized that GVHD risk depends on the model and should be considered during experimental planning.

Qg: What are the best practices for housing immunocompetent and immunosuppressed animals?

Panel response: Combined housing of immunocompetent and immunosuppressed animals is not recommended. This is due to an increased risk of severe, and often fatal, infections in immunosuppressed animals. If both categories of mice are housed together, then strict protocols are required to ensure the protection of the immunosuppressed mice. The main concern is that healthy immunocompetent animals may carry pathogens that do not cause them harm but can be lethal to immunosuppressed animals. Such pathogens can readily spread via airborne particles, through contaminated equipment, and via contact with animal handlers, for example. Whenever possible there should be separate designated rooms for each of the animal groups. For the immunosuppressed animals there should be enhanced restrictions for handling the animals, rigorous sterilization procedures, and prescribed room entry and exit protocols. All individuals handling immunosuppressed animals should wear appropriate PPE (e.g., Tyvek gowns/sleeves, gloves, face masks, hair bonnets, and shoe covers). In addition, and when possible, “maximum barriers” should be included in the facility in the form of independent air handling for the rooms. All cages, bedding, food, and water used for the immunosuppressed animals require sterilization – either through an autoclave system or by irradiation. Water can be acidified for pathogen control. Prophylactic antibiotic usage encourages antibiotic resistance. Therefore, this practice is not encouraged for pathogen control.

## Summary

In conclusion, this workshop provided an engaging and interactive forum for investigators to explore the diverse applications of humanized mouse models, with a primary focus on HIV research and the expanding relevance of these models in personalized cancer treatment. Participants appreciated the opportunity to openly discuss both promises and pitfalls of these models. This fostered a deeper understanding of how the models can be applied as well as where the models may fall short, in addressing specific research questions. A key highlight was the candid exchange around model variability, technical challenges, and the importance of selecting the right model for the right question. The collaborative atmosphere encouraged thoughtful dialogue and practical insights, making the session especially valuable for both new and experienced researchers. Such open assessments are essential for advancing the field, and the panelists and participants expressed hope that future workshops will continue to promote transparency, collaboration, and critical evaluation of these model systems in biomedical research.
